# Draft Genome Sequences of Two Epidemic OXA-48-Producing Klebsiella pneumoniae Clinical Strains Isolated during a Large Outbreak in Spain

**DOI:** 10.1128/genomeA.00026-18

**Published:** 2018-03-29

**Authors:** E. Gato, L. Álvarez-Fraga, J. A. Vallejo, S. Rumbo-Feal, M. Martínez-Guitián, A. Beceiro, M. Poza, G. Bou, A. Pérez

**Affiliations:** a Departamento de Microbiología, Instituto de Investigación Biomédica (INIBIC), Hospital Universitario A Coruña (HUAC), Universidad de A Coruña (UDC), A Coruña, Spain

## Abstract

We report here the draft genome sequences of Klebsiella pneumoniae strains Kp1803 and Kp3380 isolated during a large outbreak at A Coruña Hospital in Spain. The final genome assemblies for Kp1803 and Kp3380 comprise approximately 6.6 and 6.1 Mb, respectively, and both strains have G+C contents of 57.2%.

## GENOME ANNOUNCEMENT

The emergence of antibiotic-resistant Klebsiella pneumoniae strains makes it difficult to treat and prevent infections (1, 2) caused by this microorganism. An increasing prevalence of carbapenem-resistant K. pneumoniae strains has been reported worldwide (3–7). A Coruña Hospital experienced an outbreak of infections caused by carbapenem-resistant K. pneumoniae strains that affected more than 500 patients. These strains showed a remarkable capacity to colonize the gastrointestinal tract of patients, which acts as a reservoir for transmission and makes controlling the spread of infection difficult.

Two strains, Kp1803 and Kp3380, were isolated from the same patient with urinary tract infection in a period of 6 months. The second isolate, Kp3380, showed a reduced susceptibility to colistin compared to the first isolate, Kp1803. Here, we report the genome sequences of these two K. pneumoniae strains, which may help us to gain insight into outbreak progression despite the early implementation of infection-control procedures.

Genomic DNA was isolated using the Wizard genomic DNA purification kit (Promega) following the manufacturer’s protocols. Genome sequencing was performed using the GS Junior sequencer (454 Life Sequencing, Inc.), and the whole-genome shotgun fragment libraries were constructed from 500 ng of genomic DNA using a rapid library preparation kit. The GS Junior Titanium emulsion PCR Lib-L kit was used for the amplification of the shotgun libraries. The GS Junior Titanium sequencing system, combined with GS Junior Titanium PicoTiterPlate kits, was used to determine the nucleotide sequence of the amplified DNA libraries. Standard 454 pyrosequencing protocols were followed. Reads were assembled into contigs using the 454 gsAssembler software program with default parameters. Contigs were reordered onto the K. pneumoniae ATCC 43816 KPPR1 (GenBank accession number CP009208) and K. pneumoniae PMK1 (GenBank accession number CP008929) reference genomes using the contig-ordering tool of the Java-based graphical-interface program MAUVE (version 2.3). Genome annotations were performed using the Rapid Annotations using Subsystems Technology (RAST) server. Strain Kp1803 was sequenced twice, and the resulting libraries were combined in the same assembly. A total of 151,948 reads (68,432,817 bp), with an average length of 450.37 bp, were generated in the first sequencing, and 122,612 reads (50,334,328 bp), with an average length of 410.52 bp, were generated in the second; 98.95% and 98.61% of the reads coming from the first and second sequencings, respectively, were assembled, resulting in a total of 107 contigs, 102 of them being large contigs with lengths ranging from 643 to 349,019 bp. The average and *N*_50_ sizes of these large contigs were 54,965 bp and 124,193 bp, respectively. The estimated complete genome size was 6.6 Mb with a G+C content of 57.2% and a total of 5,331 protein-coding sequences. For strain Kp3380, a total of 183,509 reads (78,976,972 bp) were generated, with an average length of 430.88 bp; 99.40% of the reads were assembled, resulting in a total of 169 contigs, 159 of them being large contigs with lengths ranging from 613 bp to 288,531 bp. The average and *N*_50_ sizes of these large contigs were 35,128 bp and 69,019 bp, respectively. The estimated genome size was 6.1 Mb with a G+C content of 57.2% and a total of 5,381 protein-coding sequences.

### Accession number(s).

The whole-genome shotgun sequencing projects reported here have been deposited at GenBank under the accession numbers PITN00000000 for strain Kp1803 and PITM00000000 for strain Kp3380.
